# Virally Mediated Connexin 26 Expression in Postnatal Scala Media Significantly and Transiently Preserves Hearing in Connexin 30 Null Mice

**DOI:** 10.3389/fcell.2022.900416

**Published:** 2022-04-27

**Authors:** Li Zhang, Wenwen Wang, Sun Myoung Kim, Jianjun Wang, Binfei Zhou, Weijia Kong, James Zheng, Xi Lin

**Affiliations:** ^1^ Department of Otorhinolaryngology, Union Hospital, Tongji Medical College, Huazhong University of Science and Technology, Wuhan, China; ^2^ Department of Otolaryngology, Emory University School of Medicine, Atlanta, GA, United States; ^3^ Department of Cell Biology, Emory University School of Medicine, Atlanta, GA, United States

**Keywords:** connexin, scala media, cochlea, gene therapy, virus, mouse, hearing sensitivity

## Abstract

Non-sensory cells in the sensory epithelium of the cochlea are connected extensively by gap junctions. Functionally null mutations in *GJB6* (encoding Cx30) cause hearing loss in humans. In this study, we injected AAV1-CB7-*Gjb2* into the scala media between P0-2 in the cochlea of *Gjb6* ^−/−^ mice. The injection increased Cx26 expression and significantly preserved auditory functions. However, the hearing preservation gradually declined and essentially disappeared 3 months after the injections. In contrast, the morphological preservation was still significant at 3 months post-injection. We found that the expression of Cx26, at both the mRNA and protein levels, showed substantial decreases during the 3-month period. Curiously, treatments by injecting AAV1-CB7-*Gjb6* with the identical approach failed to yield any hearing preservation. Our results demonstrated the first successful cochlear gene therapy treatment in mouse models by virally expressing a companion gene of *Gjb6*.

## Introduction

Hearing loss (HL) is the most common sensory deficit in humans (Bowl et al., 2017). According to data published by the World Health Organization (WHO), around 466 million people worldwide are affected by disabling HL. Thirty-four million of the affected people are children (https://www.who.int/news-room/fact-sheets/detail/deafness-and-hearing-loss). Approximately 1 in 500 infants is affected by congenital HL and most (50–60%) of cases are caused by genetic mutations (Smith et al., 2005; Korver et al., 2017). Children with congenital HL are affected in many other aspects of their lives, including delayed language (Roberts, 1979; Kennedy et al., 2006) and cognitive developments (Roberts, 1979; Davis and Hind, 1999), difficulties in achieving better school performance (Culbertson and Gilbert, 1986; Baille et al., 1996) and in preserving their quality of life (Wake et al., 2004; Roland et al., 2016).

Gap junctions (GJs) are intercellular membrane channels that facilitate the movement of ions and biochemically active molecules among functionally coupled cells (Wu et al., 2019). Connexins (Cxs) are protein subunits used in assembling GJs, and compatible subtype of Cxs co-assemble to form homomeric or heteromeric GJs in organ/tissue specific manners (Mese et al., 2007; Goodenough and Paul, 2009). GJs co-assembled from Cx26 and Cx30 are the predominant form of GJs in the inner ear. These GJs connect all types of non-sensory cells in the organ of Corti (OC), the fibrocytes in lateral wall and basal cells in the stria vascularis (SV) (Forge et al., 2003; Wu et al., 2019). Mutations in *GJB2* (encoding Cx26) and *GJB6* (encoding Cx30) result in hereditary HL, which accounts for about half of the all cases of human hereditary HL cases (Wu et al., 2019). More than 300 deafness mutations have been reported in *GJB2* and *GJB6* (Stenson et al., 2017). One common aspect of these *GJB2* and *GJB6* mutations is that most of them generate functionally null Cxs. Both *Gjb6* ^*−/−*^ (Cx30^−/−^) (Teubner et al., 2003) and conditional *Gjb2* ^*−/−*^ (Cohen-Salmon et al., 2002; Wang et al., 2009) mice are deaf. *Gjb6* ^*−/−*^ mice exhibit severe hearing impairment and never develop the endocochlear potential (EP). Both non-sensory cells and hair cells in the cochlea degenerate after postnatal day 18 (P18) (Teubner et al., 2003). However, another Cx30 knock-out mouse model (Cx30^Δ/Δ^) generated by Boulay et al. (2013) shows normal hearing. The Cx30^−/−^model shows a very low expression of Cx26 compared to the Cx30^Δ/Δ^ model, in which half of Cx26 expression was preserved, indicating that the absence of Cx30 alone is not responsible for deafness.

Current major treatment options for sensorineural hearing loss (SNHL) patients are hearing aids and cochlear implants (CIs). However, the two options have some major limitations. Hearing aids rely on residual hearing to work. The resolution of CIs still needs improvement and is quite expensive. Tremendous effort has been spent to find novel treatments, including cochlear gene therapy, that are expected to restore hearing with more natural sound quality and to have a significantly lower lifetime cost. Rapid progress in the study of cochlear gene therapy has been made in the last few years. Most hereditary HL is caused by homozygous recessive mutations in one gene, which means genetic hearing loss is potentially amenable by exogenous expression of a single wild-type (WT) gene in many cases (Akil et al., 2012; Zhang et al., 2018). Adeno-associated virus (AAV) mediated gene expression had been used to successfully prevent hearing loss in several mouse models. Previous studies from our lab and others showed that Cx30 null mice displayed normal hearing if experimental manipulations maintain cochlear Cx26 protein level at the WT level (Ahmad et al., 2007; Boulay et al., 2013). In this study, we injected AAV1 vectors to express either *Gjb2* or *Gjb6* in the scala media (SM) of *Gjb6* ^*−/−*^ mice. Our goal is to investigate whether supplemental viral expression of either Cx26 or Cx30 at the neonatal stage could preserve the auditory functions. Results showed successful hearing preservation in *Gjb6* ^*−/−*^ mice by our current approach performed at early postnatal stage for about 3 months only with the overexpression of AAV1-mediated Cx26 expression.

## Materials and Methods

### Design and Packaging of Recombinant AAV1 Constructs

Full-length cDNA of *Mus musculus Gjb2* was obtained by enzymatic digestion from the pCMV6-Kan/Neo-*Gjb2* plasmid (OriGene Technologies Inc., Rockville, MD) by using KpnI and XhoI. The plasmid of pCMV6-Kan/Neo-*Gjb6* was also purchased from OriGene Technologies. The GFP gene was obtained from the pAAV-GFP vector (Cell Biolabs Inc., San Diego, CA) by digesting with EcoRI and SalI. The cut-off cDNAs were inserted into the pENN.AAV.CB7.CI.RBG (Gene Therapy Program, University of Pennsylvania) to form the plasmids of either the pAAV-CB7-*Gjb2,* pAAV-CB7-*Gjb6* or pAAV-CB7-GFP (Supplementary Figure S1B), respectively. AAV1 virus particles were prepared at the Emory Viral Vector Core (http://neurology.emory.edu/ENNCF/viral_vector/). More details about the design and construction of viral plasmids (Yu et al., 2014) and virus production (Wang et al., 2013) were described previously. Virus titers were measured by a method based on a real-time PCR method (Aurnhammer et al., 2012). The titers of AAV1-CB7-*Gjb2,* AAV1-CB7-*Gjb6* and AAV1-CB7-GFP were typically 1.5 × 10^12^, 4.5 × 10^12^ and 1.5 × 10^13^ genome copies/ml, respectively. The virus aliquots (10 μl each, in phosphate buffered solution (PBS)) were stored in −80°C freezer. One aliquot was thawed at room temperature prior to the injection.

### Mouse Models and Virus Injection Procedures

Animal care and use were in accordance with the guidelines of the National Institutes of Health (NIH) and the protocol was approved by the Institutional Animal Care and Use Committee of the Emory University. Surgical procedures of virus injection were performed following the same protocol as those previously described (Chang et al., 2015). *Gjb6* ^*−/−*^ mice were purchased from the European Mouse Mutant Archive (EMMA, www.infrafrontier.eu) and were used in our previously published studies (Ahmad et al., 2007; Chang et al., 2008; Sun et al., 2009). *Gjb6* ^*−/−*^ mice were originally generated in a mixed 129P2/OlaHsd and C57BL/6NCrl strain background (Teubner et al., 2003). Mice with the CBA/CaJ background, unlike the C57BL/6 background, have consistently displayed minimal age-related HL (Zheng et al., 1999; Sun et al., 2009). In order to minimize the risk of early-onset age-related HL due to genetic background, we backcrossed the purchased *Gjb6* ^*−/−*^ mice with the CBA/CaJ mice (purchased from the Jackson Lab., Bar Harbor, ME) for more than 30 generations and obtained a near homogeneous CBA/CaJ genetic background for *Gjb6* ^*−/−*^ mice used in this study.

Recombinant AAV1 vectors were injected into the left ear of *Gjb6* ^*−/−*^ mice from P0 to P2. The ABRs were recorded (Ahmad et al., 2007) at 1, 2, and 3 months post-injection. The mouse cochleae were extracted for measuring viral DNA copies, *Gjb2* mRNA and Cx26 protein levels. A diagram showing the experimental design is given in Supplementary Figure S1A. Mice were anesthetized by placing them on ice. A skin incision was made behind the ear to expose the otic bulla. The tympanic membrane and auditory ossicles were used as landmarks during surgery. The locations of the basal cochlear turn and round window membrane (RWM) were identified by their anatomical relation to the stapedius artery. Glass micropipettes (tip size 10–15 μm) were made on a horizontal pipette puller (Model P-2000, Sutter Instrument, Novato, CA). We used a micromanipulator (MP-285, Sutter Instrument, Novato, CA) to precisely control the movement of injection pipettes. The tip of the pipette was advanced to penetrate into the SM or scala tympani (ST) to inject ∼1.0 µl of virus fluid into the left cochlea by using a Picospritzer III pressure microinjection system (Picospritzer III, Parker Hannifin Corp, NY). Fast green dye (Sigma-Aldrich Inc., St Louis, MO), which was visible when observed under bright light illumination of a dissection microscopy (Stemi 2000, Zeiss, Germany), was included in the solution to help visually monitor and confirm the fluid ejections. After surgeries, mice were allowed to recover on a 37°C heating pad (model TR-100, Fine Science Tool Inc., Foster City, CA) before returning to the animal housing facility.

### Measurement of the Auditory Brainstem Responses

Mice were anesthetized with the mixture of ketamine (80 mg/kg), xylazine (10 mg/kg) and acepromazine (30 mg/kg). Tone burst (4–32 kHz) or click sound stimuli generated by Biosig were delivered by a speaker (model MF1, Tucker Davis Technologies, Alachua, FL) to the ear through a 1/8′ I.D. PVC tubing. Speakers are calibrated every month by following manufacturer’s instructions. ABRs from WT mice with or without AAV1-CB7-GFP injections and *Gjb6* ^*−/−*^ mice injected with or without AAV1-CB7-*Gjb6* were measured at 1 month after the injection. ABRs in *Gjb6* ^*−/−*^ mice with or without AAV-CB7-*Gjb2* injection were measured at 1, 2, and 3 months post-injection (Supplementary Figure S1A). More details for ABR test protocols and determination of ABR thresholds can be found in our published papers (Ahmad et al., 2007).

### Immunolabelling of Cochlear Whole Mount Preparations

The cochleae of anesthetized mice were dissected from mice heads. Samples were fixed with 4% paraformaldehyde (PFA) in PBS for 2 h. Cochleae obtained from animals older than 10 days were decalcified with 10% EDTA for 4 days until the bony tissue was soft to the touch of forceps. The Cochlea was dissected out by carefully removing the bony tissue surrounding it. The Reissner’s membrane and tectorial membrane were removed, and the OC and the lateral walls were preserved. Samples were blocked and permeabilized with 10% goat serum in 0.1% Triton X-100 in PBS (PBST) at room temperature for 1 h. They were then incubated with primary antibodies against either Cx26 (1:200, Invitrogen, 71–0500, Waltham, MA), Cx30 (1:200, Invitrogen, 71–2200, Waltham, MA), Myosin Ⅶa (1:200, *Proteus* biosciences Inc., 25–6,790, Ramona, CA) or GFP (1:200, Rockland Immunochemicals Inc., 600–401–215, Limerick, PA) diluted with 0.1% PBST at 4°C overnight. After washing three times with PBS (10 min/each wash) at room temperature, samples were incubated with either Alexa 488 conjugated secondary antibodies (1:500, Invitrogen, A11034, Waltham, MA) or Phalloidin-Tetramethylrhodamine B isothiocyanate (Phalloidin-TRITC) (1:1,000, Sigma-Aldrich Inc., P1951-.1 MG, St Louis, MO) for 1 h at 4°C. Processed samples were washed for three times in PBS (10 min/each wash) at room temperature. Samples were mounted in fluoromount-G antifading solution and examined using a Zeiss LSM 510 confocal microscope (Carl Zeiss, Oberkochen, Germany). More details can be found in our published papers (Chang et al., 2015).

### Measurement of Viral Titer, RNA and Protein Levels of the Cx26 in the Cochlea

Genomic DNA and RNA were extracted from the cochleae at time points of 1, 2, and 3 months post-injection (Supplementary Figure S1A). DNA and RNA were isolated using AllPrep DNA/RNA mini kit (Qiagen, 80,204, Hilden, Germany) from the cochleae. RNA was treated by DNase I (Promega, M6101, Madison, WI) first and then used for cDNA synthesis using qScript cDNA SuperMix (Quantabio, 95,048, Beverly, MA). Semi-quantitative PCR analysis was performed using the following primers: CMV-F 5′ACG​GGG​TCA​TTA​GTT​CAT​AGC3′ and CMV-R 5′GCT​CAC​CTC​GAC​CAT​GGT​AA3′ for viral DNA; *Gapdh*-F 5′GCT​GAG​TCA​TGG​TGG​TTC​TG3′ and *Gapdh*-R 5′TTC​CAA​CCC​TAA​TCC​CAG​GG3′ for *Gapdh* mRNA; *Gjb2*-F 5′TTC​AGA​CCT​GCT​CCT​TAC​CG3′ and *Gjb2*-R 5′ATG​CAT​AGC​TAC​CAG​GAG​GG3′ for *Gjb2* mRNA; β-actin-F 5′GGG​ACC​TGA​CGG​ACT​ACC​TC3′ and β-actin-R 5′CCA​TAC​CCA​AGA​AGG​AAG​GCT3′ for β-actin mRNA.

Total protein was extracted from the cochleae at time points of 1, 2, and 3 months post-injection. Cochlear samples were homogenized by a pestle in a 1.5 ml microcentrifuge tube and cochlear lysate was prepared using RIPA buffer (150 mM NaCl, 1.0% Triton X-100, 0.5% sodium deoxycholate, 0.1% SDS, 50 mM Tris-pH 8.0) containing protease inhibitor cocktail (Roche, 04693116001, Indianapolis, IN). The lysate was passed through a 22-gauge needle with syringe at least 10 times prior to centrifugation and processed for standard western blot with mouse anti-Cx26 antibody (0.25 µg/ml, Invitrogen, 71–0500, Waltham, MA), and mouse anti-α-tubulin antibody (0.25 µg/ml, Santa Cruz Biotechnology, sc-32293, Dallas, Texas).

### Statistical Analysis

Data were statistically analyzed using *t*-test and they are presented as mean ± standard deviation (SD). The significance level in statistical analyses was set at *p <* 0.05. Statistical significance is indicated in the figures as follows: n.s., not significant; *p < 0.05; **p < 0.01; ***p < 0.001.

## Results

### Virally Mediated Gjb2 Expression Significantly Enhanced Cx26 Protein Level and the Formation of GJs in Supporting Cells

We first optimized the viral transduction efficiency by comparing results of AAV1-CB7-GFP (green fluorescent protein) expressions obtained from injections into either the SM or ST of WT mice at P0 to P2. The signal of GFP, 1 week after injection, was examined from immunolabelling of whole mount cochlear samples. GFP-positive cells were identified in inner hair cells (IHCs), outer hair cells (OHCs), Hensen’s cells (HeCs), inner sulcus cells (ISCs), outer sulcus cells (OSCs), Claudius cells (CCs) and marginal cells (MCs) (Supplementary Figure S2A). Transduction efficiency achieved by injection into the SM appeared to be significantly better for most cochlear cell types lining the walls of the SM, including the targeted cochlear supporting cells (e.g., HeCs, ISCs, OSCs, CCs) (Supplementary Figures S2A,B). In addition, tone-burst auditory brainstem response (ABR) tests showed that injections into the SM between P0-2 did not impair the hearing thresholds of WT mice measured at 1 month post-injection (Supplementary Figure S2C). We therefore injected viral solution into the SM in rest of our studies for the treatment of *Gjb6* ^*−/−*^ mice in order to optimize virally-mediated Cx expressions in cochlear cells targeted for treatment.

**FIGURE 1:**
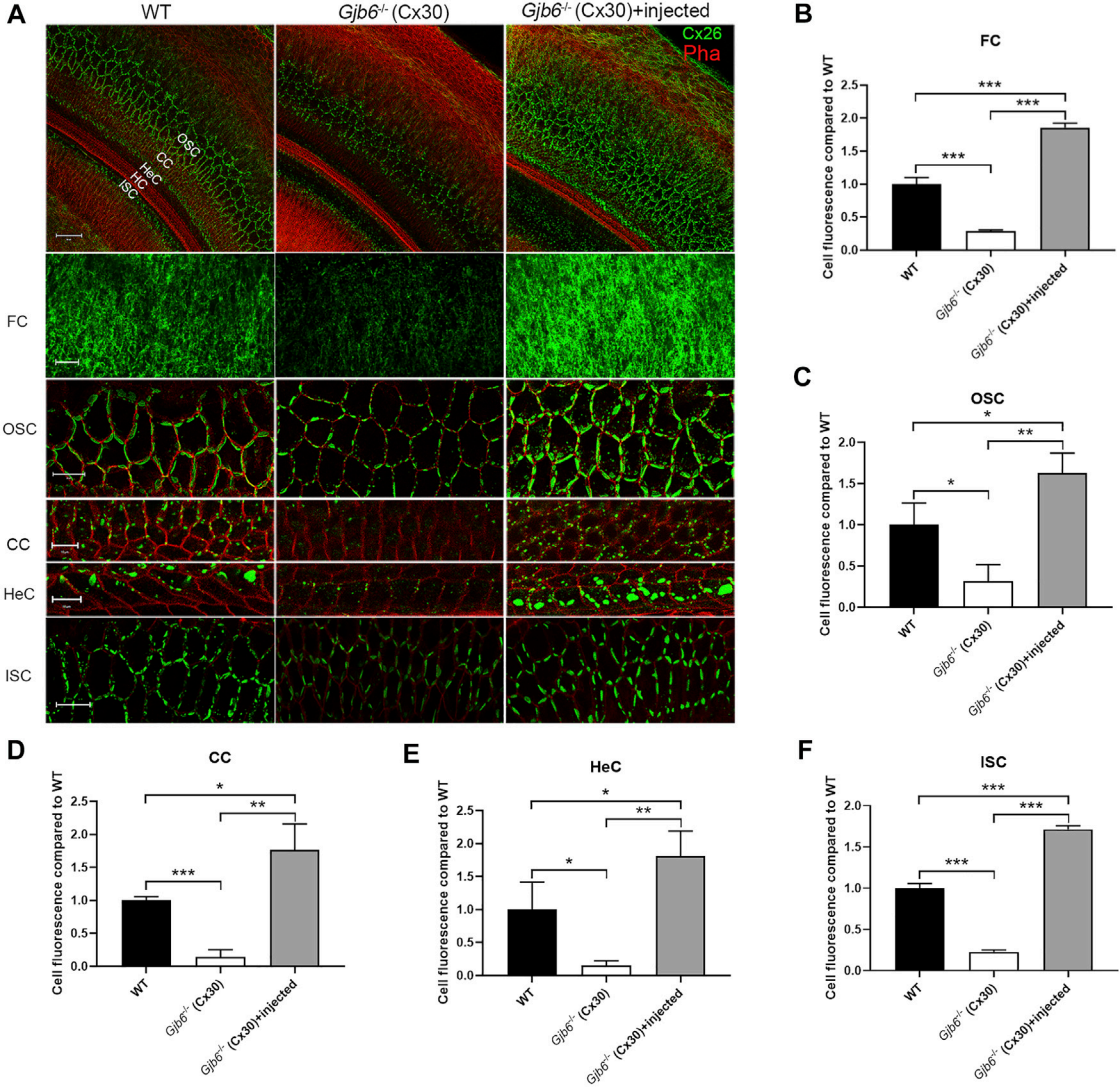
Injection of AAV1-CB7-*Gjb2* increased the formation of GJs in FCs, OSCs, CCs, HeCs and ISCs in *Gjb6* ^*−/−*^ mice. **(A)** Immunolabelling of Cx26 in WT, *Gjb6* ^*−/−*^ cochlea without or with AAV1-CB7-*Gjb2* injection. The first row: Low magnification views of representative confocal images of the C×26 expression in WT, *Gjb6* ^*−/−*^ cochlea without injection and *Gjb6* ^*−/−*^ cochlea with AAV1-CB7-*Gjb2* injection. The second to the last row: High magnification views of C×26 expression in FC in spiral ligament, OSC, CC, HeC and ISC in WT (panels in left column), *Gjb6* ^*−/−*^ cochlea without injection (panels in the middle column) and *Gjb6* ^*−/−*^ cochlea with AAV1-CB7-*Gjb2* injection (panels in the right column). The first to the fifth row were from P9 mice and the last row were from mice at 1 month post-injection. **(B–F)** Quantification of the immunofluorescence signal in FC, OSC, CC, HeC and ISC. FC, fibrocytes; OSC, outer sulcus cell; CC, Claudius cell; HeC, Hensen’s cell; ISC, inner sulcus cell; HC, hair cell; Pha, phalloidin; Cx26, connexin 26. Scale bar: 50 µm (the first row), 20 µm (the second, third and sixth row), 10 µm (the fourth and fifth row). Error bars in B-F represent standard deviation of the mean. *n* = 3 for each group, * indicates *p* < 0.05, ** indicates *p* < 0.01, *** indicates *p* < 0.001.

We performed whole mount immunolabelling to examine the cellular expression pattern of Cx26 in WT and *Gjb6* ^*−/−*^ mice with or without AAV1-CB7-*Gjb2* injections. Cx26 was widely expressed in supporting cells of the OC in WT mice (left column of Figure 1A), and the Cx26 expression was not found in the IHCs, OHCs after the AAV1-CB7-*Gjb2* injection (Figure 1A). The overall Cx26 expression level appeared to be decreased in the cochlea of un-injected *Gjb6* ^*−/−*^ mice (Figure 1), which is consistent with previous reports of others and our lab (Lautermann et al., 1998; Ahmad et al., 2003; Ahmad et al., 2007). These reduced immunoreactivities of Cx26 were observed in the fibrocytes, OSCs, CCs, HeCs and ISCs (Figure 1) in the un-injected cochlea of *Gjb6* ^*−/−*^ mice compared to those of WT. After injecting AAV1-CB7-*Gjb2* into the cochlea of *Gjb6* ^*−/−*^ mice, we observed that the Cx26 immunolabelling signal in these cells were apparently higher than the un-injected cochlea (Figure 1). The Cx26 expression was not found in the IHCs, OHCs in the cochlea with the AAV1-CB7-*Gjb2* injection (Figure 1). Western blot showed that the overall Cx26 expression levels in the cochlea of un-injected and injected *Gjb6* ^*−/−*^ mice was 39.3 ± 8.6% and 179.9 ± 30.6% respectively, when compared to the WT mice examined 1 month post-injection (left three columns of Figure 4A). The western blot result was consistent with that of whole mount immunelabelling (Figure 1). These results showed that AAV1-CB7-*Gjb2* significantly increased Cx26 protein level in the cochlear cells targeted for treatment.

### ABR Thresholds Were Preserved Temporarily for Three Months by AAV1-CB7-Gjb2 Injections While the Preservation of the Cochlear Morphology Seemed to Last Longer

**FIGURE 2:**
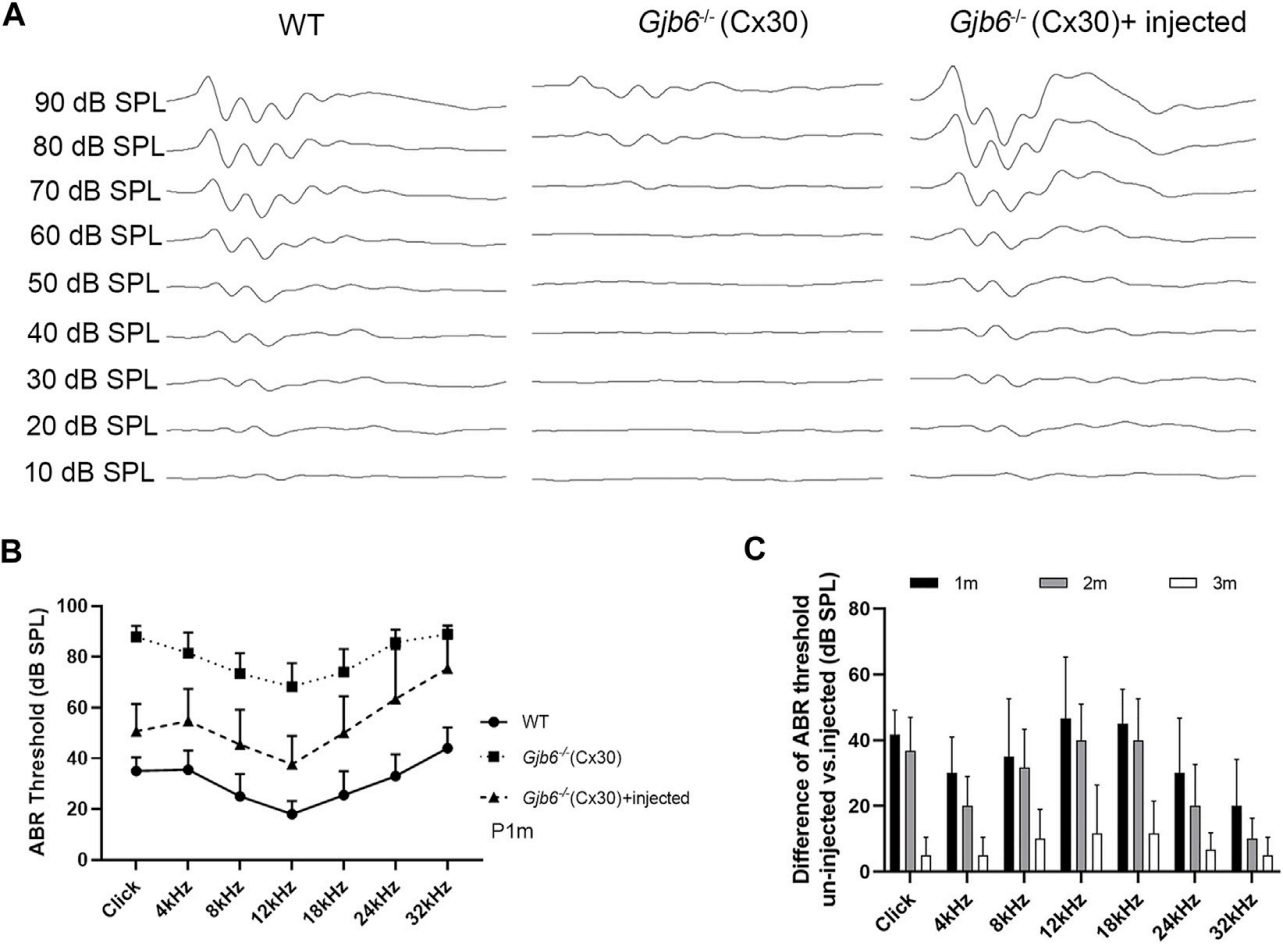
Injection of AAV1-CB7-*Gjb2* significantly preserved ABR thresholds in *Gjb6* ^*−/−*^ mice transiently. **(A)** Waveforms of ABRs recorded at 1 month post-injection from (as labeled) WT, un-injected *Gjb6* ^*−/−*^ mouse (data traces in the middle column) and *Gjb6* ^*−/−*^ mouse injected with AAV1-CB7-*Gjb2* (data traces in the right column). ABRs were recorded using 18 kHz tone bursts at sound pressure levels as indicated at the left of data traces in 10 dB steps ranging a low of 10 to the highest 90 dB SPL. **(B)** Summary of averaged ABR thresholds tested at 1 month post-injection by click sound, and by tone bursts (presented from 4 to 32 kHz) for three groups of mice: WT (filled circles, *n* = 14), un-injected *Gjb6* ^*−/−*^ (filled square, *n* = 35) and *Gjb6* ^*−/−*^ mice injected with AAV1-CB7-*Gjb2* (filled triangles, *n* = 36). Error bars represent the standard deviation of the mean. **(C)** Time course of changes in the difference in ABR thresholds between the injected and un-injected ears as tested by click and by tone bursts, as measured at 1, 2 and 3 months post-injection (labels given at the top of panel). Error bars in **(B,C)** represent the standard deviation of the mean.

To evaluate the treatment efficacy on the hearing threshold, we measured ABR thresholds using either click or tone-burst evoked responses from three groups of mice: 1) WT mice, 2) *Gjb6* ^*−/−*^ mice with the AAV1-CB7-*Gjb2* injections, from both un-injected and injected ears, 3) *Gjb6* ^*−/−*^ mice injected with AAV1-CB7-*Gjb6*. Untreated *Gjb6* ^*−/−*^ mice showed severe HL (*n* = 35, Figure 2B; data points showed by filled squares) in the range of about 60–90 dB when measure 1 month post-injections. We found that the ABR thresholds of mice treated with AAV1-CB7-*Gjb2* were significantly preserved, as measured by either click (*n* = 14) or tone-burst sounds (*n* = 35, Figure 2B; data points showed by filled triangles). The differences in ABR thresholds between the un-injected and injected ears (in the same animal) were statistically significant (Figure 2B). The average difference of ABR thresholds between the two ears evoked by click sound was 37 ± 9 dB (*n* = 14, *t*-test, *p* < 0.001). The average improvements measured by tone-bursts presented at 4, 8, 12, 18, 24, 32 kHz were 27 ± 11, 28 ± 15, 31 ± 15, 24 ± 16, 22 ± 20 and 13 ± 16 dB, respectively (*n* = 35, *t*-test, *p* < 0.001 for all frequencies).

We monitored long-term treatment efficacy in the injected *Gjb6* ^*−/−*^ mice for 3 months (*n* = 6) and found that the differences between un-injected and injected ears significantly and gradually decreased (Figure 2C). The differences in ABR thresholds measured at 1 month post-injection were 42 ± 8 dB tested by the click and were 30 ± 11, 35 ± 18, 47 ± 19, 45 ± 10, 30 ± 17 and 20 ± 14 dB at 4, 8, 12, 18, 24 and 32 kHz, respectively tested by tone bursts. The difference between ABR thresholds measured at 2 months post-injection decreased to 37 ± 10 dB tested by the click sound and 20 ± 9, 32 ± 12, 40 ± 11, 40 ± 13, 20 ± 13 and 10 ± 6 dB at 4, 8, 12, 18, 24 and 32 kHz tested by tone bursts. These differences were still statistically significant (*n* = 6, *t*-test, *p* < 0.05 for all frequencies). Measured at 3 months post-injection, these differences between treated and untreated ears were no longer statistically significant (*n* = 6, *t*-test, *p* > 0.05). We also injected AAV-CB7-*Gjb6* into the SM of the left cochlea of *Gjb6* ^*−/−*^ mice at P0-2 using the same procedures as those used to inject AAV-CB7-*Gjb2*. The expressions of Cx30 in the cochlea after injection of AAV-CB7-*Gjb6* were confirmed (Supplementary Figure S3A). However, we never observed preservation of the hearing threshold tested from more than one hundred *Gjb6* ^*−/−*^ mice (Supplementary Figure S3B).

**FIGURE 3:**
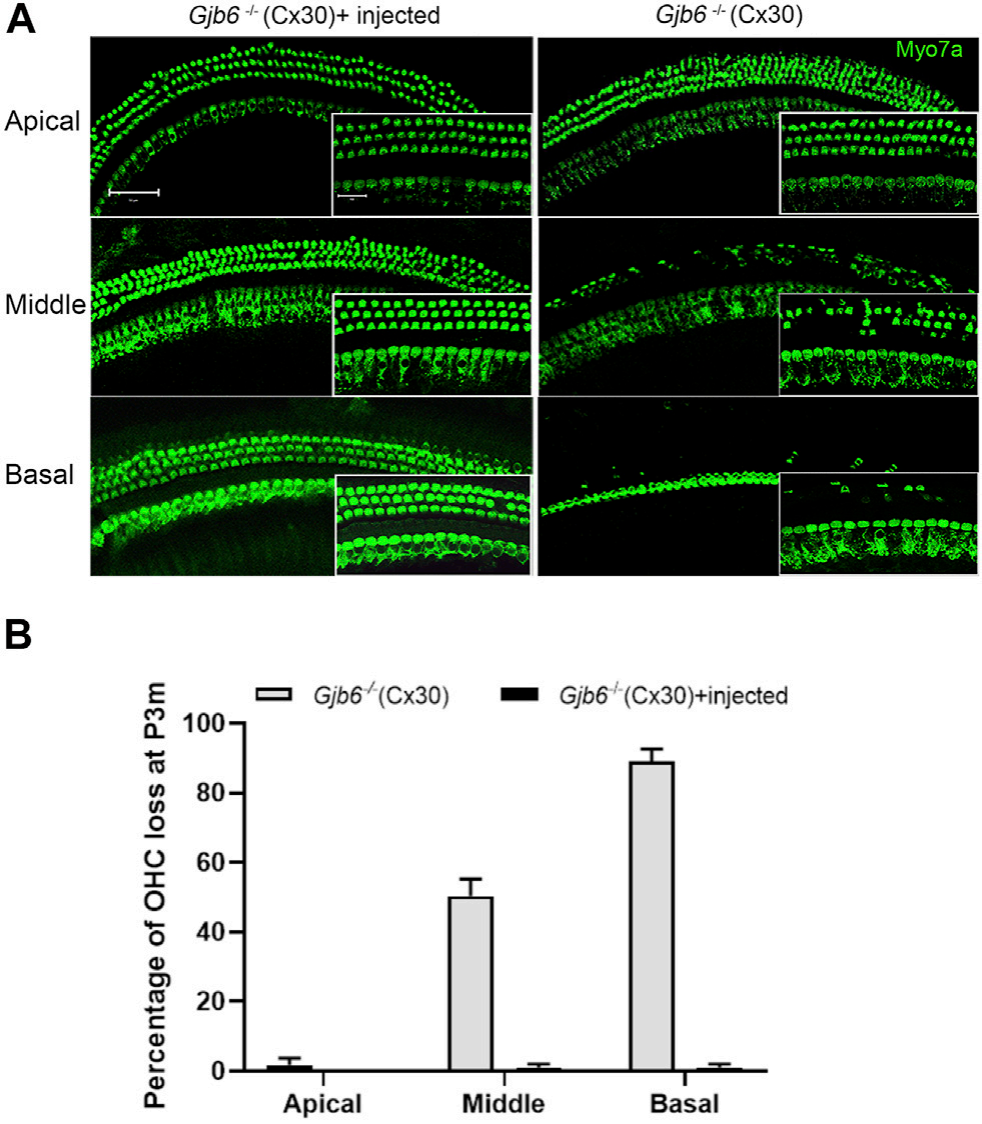
Injection of AAV1-CB7-*Gjb2* improved OHC survival in *Gjb6* ^*−/−*^ mice. **(A)** Comparison of the loss of OHCs between un-injected and injected ears in *Gjb6* ^*−/−*^ mice at 3 months post injection. Images were obtained from whole-mount cochlear samples. Insets are magnified images. **(B)** Quantifications of the percentage of OHC loss (normalized to age-matched WT controls) in the apical, middle and base turns of un-injected *Gjb6* ^*−/−*^ mice and *Gjb6* ^*−/−*^ mice injected with AAV1-CB7-*Gjb2* examined at 3 months post-injection. Error bars in **(B)** represent standard deviation of the mean (*n* = 3 for each group). Myo7a, Myosin 7A.Scale bar: 50 µm (low magnification), 20 µm (high magnification).

The morphological changes in the cochlea of *Gjb6* ^*−/−*^ mice started from the degeneration of the OHCs (Teubner et al., 2003; Sun et al., 2009). We performed immunolabelling to examine whether the injection of AAV1-CB7-*Gjb2* preserved normal morphology of OHCs. Examined at 3 months when the difference of ABR thresholds between the un-injected and injected ears were small and no longer statistically significant (Figure 2C), we found severe OHCs degeneration in both the middle (50.33 ± 4.93% loss of OHCs) and basal turns (89.00 ± 3.61%) of the un-injected cochlea (right column of Figures 3A,B). In contrast, the middle and basal turn in the injected cochlea showed relatively few OHC loss (<1%) (left column of Figures 3A,B). These results suggested that morphological preservation (e.g., survival of OHCs) after injection of AAV1-CB7-*Gjb2* lasted longer than the hearing preservation in *Gjb6* ^−/−^ mice.

### Time Course of the Changes in the Viral Titer, mRNA and Protein Levels of Cochlear Gjb2

**FIGURE 4:**
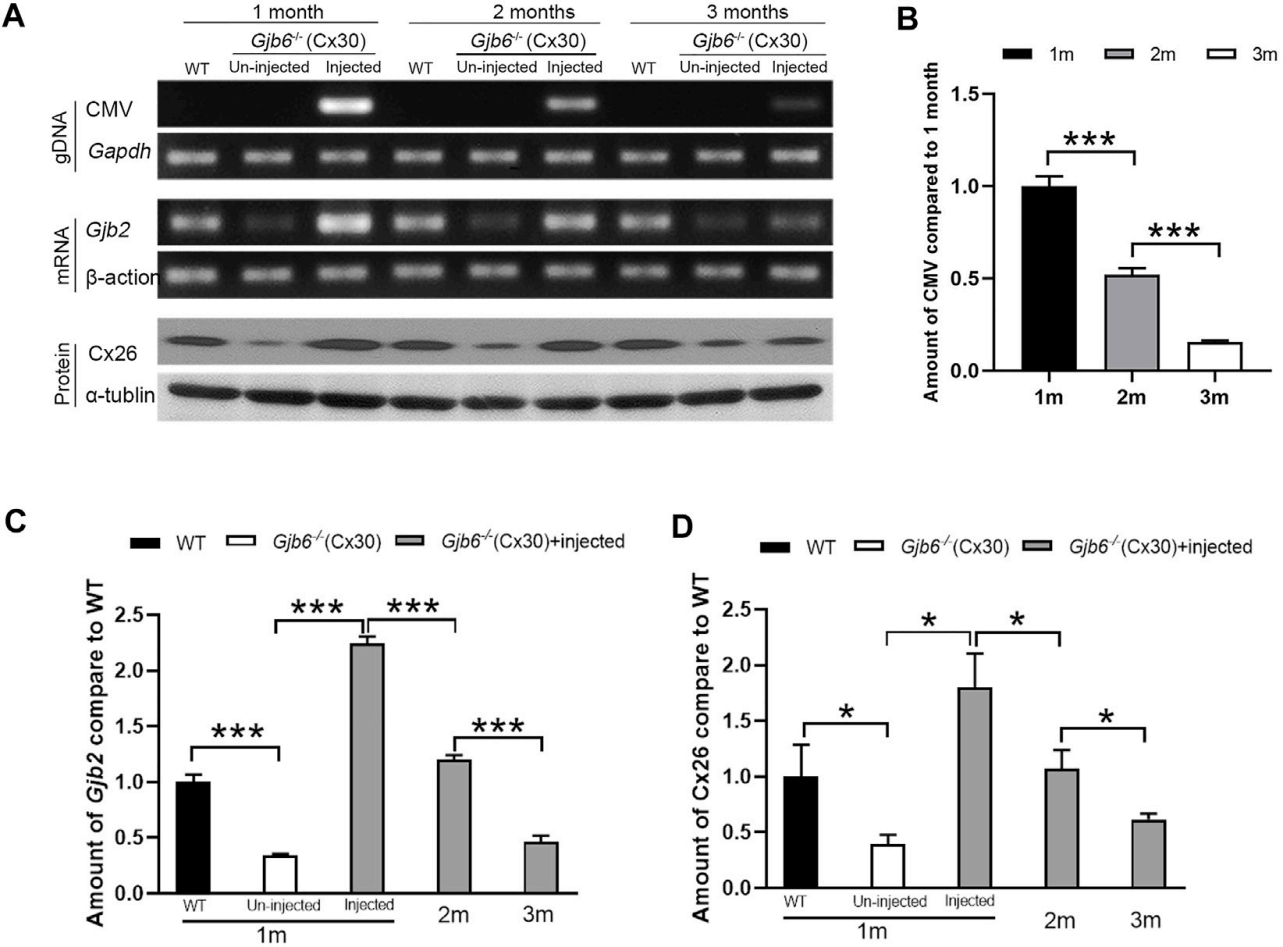
Viral titer, *Gjb2* expression at the mRNA and protein levels all decreased gradually in *Gjb6* ^*−/−*^ mice during the 3 month period after injection with AAV1-CB7-*Gjb2*. **(A)** Representative data showing the levels of AAV1-CB7-*Gjb2* genome copy numbers (top rows), *Gjb2* mRNA (middle rows) and Cx26 protein levels (bottom rows) measured from (as labeled in A) WT, un-injected *Gjb6* ^*−/−*^ mice and *Gjb6* ^*−/−*^ mice injected with AAV1-CB7-*Gjb2* at 1, 2 and 3 months post-injection. *Gapdh*, β-actin and α-tubulin were used as loading controls for AAV genome, *Gjb2* mRNA and Cx26 protein, respectively. **(B)** Densitometric quantification of AAV genome **(C)** Densitometric quantification of *Gjb2* mRNA level from three groups of mice as labelled. **(D)** Densitometric quantification of Cx26 protein level as measured by western blots. Error bars in **(B–D)** represent standard deviation of the mean. *n* = 3 for each group, * indicates *p* < 0.05, *** indicates *p* < 0.001.

To investigate the underlying mechanism for decreased treatment efficacy (Figure 2C) over a relatively short period of 3 months, we examined the changes in the levels of AAV genome copy numbers (viral titer), *Gjb2* transcripts (mRNA), and Cx26 protein levels in the cochleae. Primers specific for the CMV promoter amplified the AAV genome (Supplementary Figure S1C) and the AAV was detected only in the injected cochleae (top rows of Figure 4A). The copy numbers of AAV genome were significantly decreased gradually during the 3-month period (top rows of Figures 4A,B). The AAV genome copy numbers measured at second and third month were 51.9 ± 3.4% and 15.5 ± 0.8% of that of the first month, respectively (Figure 4B). For the *Gjb2* mRNA level*,* un-injected cochlea was 34.0 ± 1.7% of that of the WT, while the injected cochlea was 224.5 ± 6.3% compared to the WT cochlea when examined at 1 month. The mRNA levels decreased dramatically during the 3 months period after injection (middle rows of Figures 4A,C). They were 120.0 ± 4.2% and 46.3 ± 5.7% of the WT levels measured at two and 3 months post-injection, respectively (Figure 4C). We performed western blots to examine the Cx26 protein level in the cochlea. The Cx26 protein in the untreated cochlea was 39.3 ± 8.6% compared to the WT mice when measured at 1 month. The Cx26 protein level in the injected cochlea was 179.9 ± 30.6% of the WT cochlea measured at 1 month post-injection. The protein levels of the injected cochlea gradually decreased (bottom rows of Figures 4A,D). They were 107.2 ± 16.9% and 61.6 ± 5.2% of the WT level when measured at two and 3 months post-injection, respectively (Figure 4D).

## Discussion

In this study we injected either AAV1-CB7-*Gjb2* or AAV1-CB7-*Gjb6* to the SM of *Gjb6* ^−/−^ mice by the same procedure. Viral titer of AAV1-CB7-*Gjb6* was no less than the AAV1-CB7-*Gjb2*. However, the hearing thresholds of *Gjb6* ^−/−^ mice were only preserved by postnatal injection (P0-2) of the AAV1-CB7-*Gjb2* (Figure 2). Our published studies found that Cx26 plays a unique and dominate role in the early development of the cochlea (Qu et al., 2012). We also showed that transgenic mice with extra copies of the *Gjb2* expressed from the bacteria artificial chromosome in the *Gjb6* ^−/−^ mice have normal hearing (Ahmad et al., 2007). Boulay et al. (2013) reported that the hearing loss phenotype exhibited by *Gjb6* ^*−/−*^ mice depends on the cumulative effect of deletion of Cx30 and 3’ insertion of the lacZ and neo genes, which causes dramatic reduction in Cx26 protein level in the cochlea of *Gjb6* ^*−/−*^ mice. These data support that the reduced Cx26, other than the deletion of Cx30, is the reason for the hearing loss in *Gjb6* ^*−/−*^ mice. They further support our hypothesis that virally increasing the Cx30 in the absence of appropriate Cx26 level is unlikely to preserve hearing in *Gjb6* ^*−/−*^ mice. The current study supports the hypothesis that up-regulation of Cx26 other than Cx30 is the key in preventing deafness caused by Cx30 null mutation. Comparing to prior cochlear gene therapy studies, one important and novel design in our study was that we virally expressed the *Gjb2,* which is a companion gene of the *Gjb6* that displayed positive treatment outcomes in mice*.* The new design expanded the design principles of current gene replacement/supplement therapies, and support the importance of studying gene-gene interactions as the basis for mechanism-based gene therapy in the future.

Several studies have been published with the aim of preserving hearing in Cx30 null mice. Crispino et al. (2017) failed to preserve the auditory function by injecting BAAV-Cx26 or BAAV-Cx30 to *Gjb6* ^−/−^ mice via semicircular canal at P4. The results were consistent with our observation that directly supplementing Cx30 in the early postnatal age failed to preserve hearing in *Gjb6* ^−/−^ mice. However, delivering BAAV-Cx26 also failed to restored hearing in that study. The reason may be due to a rapid decrease in the transcript level of Cx26, which measured only 15.5 ± 4.5% (*p* < 0.005, ANOVA) of its initial peak 30 days after injections. In this study, the transcription of Cx26 could be maintained at the WT level for about 2 months (Figure 4). Miwa et al. (2013) performed electroporation-mediated transuterine gene transfer of the WT Cx30 gene into the otocysts of homozygous Cx30 null mice. They reported significant hearing preservation. The Cx26 level was not measured in that study, therefore it is unclear whether the observed hearing preservation was directly caused by embryonic boost in the Cx30 expression or indirectly by increasing in Cx26 protein level when the Cx30 level is boosted in the embryonic stage.

Another important finding was that the treatment efficacy only lasted for less than 3 months by our current injection protocols. A survey of published cochlear gene therapy studies (Supplementary Table S1) revealed that at least half of the prior studies showed relatively short efficacy of less than 6 months in mouse models regardless of injections made at embryonic, neonatal or later stages (Chang et al., 2015; Kim et al., 2016; Zhang et al., 2018; Yoshimura et al., 2019). For examples, an *in-utero* virus-mediated gene therapy approach preserves the hearing of *MsrB3* ^−/−^ mice. However, hearing thresholds begin to increase at 7 weeks of age (Kim et al., 2016). Chang et al. (2015) showed that restored hearing in *Kcnq1* ^−/−^ mice by delivering AAV-*Kcnq1 via* injecting into the SM at P0-2 started to deteriorate beginning at 18 weeks. Hearing restoration of *Tmc1* ^Bth/+^ mice using a single intracochlear injection of an artificial microRNA carried by an AAV vector at adult stage also showed transient efficacy and rescued hearing start to decline at 8 weeks after injections (Yoshimura et al., 2019). The transient efficacy of gene therapies was also observed in gene therapy studies of other organs. Administration of AAV-G6Pase into dogs with GSD-Ia prevented hypoglycemia, but the treatment efficacy waned between 2 and 30 months of age (Demaster et al., 2012). In human follow-up studies of ocular gene therapy in the LCA2 clinical trials, the vision improvement in LCA patients by AAV2-RPE65 injections progressively diminished after 3 years (Bainbridge et al., 2015; Jacobson et al., 2015). These studies suggest that the long-term treatment efficacy of cochlear gene therapy is not guaranteed. One popular hypothesis for the efficacy decline is that the expression level of virally-expressed gene may slowly decline to below a needed therapeutic threshold level (Bainbridge et al., 2015).

To directly investigate the underlying mechanism for decline in treatment efficacy, we measured the time course of changes in the viral titer, the expression of Cx26 at both the mRNA and protein levels, and found that all of which declined significantly during the 3-month period in the cochlea. At 3 months post-injection when the hearing of injected and un-injected ears had no statistically significant differences, we found that both total mRNA and protein levels of Cx26 were below those of the WT levels (Figure 4). These results suggested that the maintenance of normal cochlear functions needed the Cx26 to be expressed above a certain threshold level, below which the cochlear functions cannot be maintained. Cunningham et al. (2008) and Yiu et al. (2010) both showed that the rapid decline of AAV-mediated hepatic gene expression was resulted from the fast rate of the hepatocellular cellular turnovers. As the supporting cells in the OC, the cells in SV and lateral wall may turnover slowly (Dunaway et al., 2003; Lang et al., 2003; Li et al., 2017), we suspect that this may be one reason that the Cx26 expression gradually declined. How to maintain the long-term efficacy is a critical issue for future clinical translation the cochlear gene therapy. To extend the treatment efficacy, we postulate that a second injection or even more follow-up injections at the adult stage may be needed. Demaster et al. and Young et al. show that re-administration of the AAV vector could prolong the treatment efficacy in dogs with GSD-Ia (Demaster et al., 2012; Lee et al., 2018). For the *Gjb6* ^*−/−*^ mice, the second injection to prevent the decline of hearing appears to be possible since most of the hair cells in the treated ears did not degenerate at 3 months (Figure 3). Another approach may be to discover more viral vectors with higher transduction efficiency or vectors that integrate into the genome. Geng et al. (2017) showed that treatment efficacy of gene therapy in *Clrn1* KO-TgAC1 mice with AAV-*Clrn1*-UTR injection could last for 150 days while mice treated with AAV-*Clrn1* could only last for 90 days. These studies suggest that designs to include more appropriate promoters or tags may potentially increase long-term efficacy of the gene therapy.

In summary, our study showed that virally mediated *Gjb2* expression could significantly prevent hearing loss in *Gjb6* ^−/−^ mice for about 2 months. The preservation of cochlear morphology is longer than the hearing preservation. Our studies also suggested research directions and technical issues for future studies. One of them is to improve long-term efficacy such that cochlear gene therapy may have a better chance and appeal for successful clinical translation into human applications.

## Data Availability

The original contributions presented in the study are included in the article/Supplementary Material, further inquiries can be directed to the corresponding author.
