# Corrigendum: Virally mediated connexin 26 expression in postnatal scala media significantly and transiently preserves hearing in connexin 30 null mice

**DOI:** 10.3389/fcell.2022.969989

**Published:** 2022-07-18

**Authors:** Li Zhang, Wenwen Wang, Sun Myoung Kim, Jianjun Wang, Binfei Zhou, Weijia Kong, James Zheng, Xi Lin

**Affiliations:** ^1^ Department of Otorhinolaryngology, Union Hospital, Tongji Medical College, Huazhong University of Science and Technology, Wuhan, China; ^2^ Department of Otolaryngology, Emory University School of Medicine, Atlanta, GA, United States; ^3^ Department of Cell Biology, Emory University School of Medicine, Atlanta, GA, United States

**Keywords:** connexin, scala media, cochlea, gene therapy, virus, mouse, hearing sensitivity

In the published article, there was an error regarding the **author list** contributions. Li Zhang, Wenwen Wang and Sun Myoung Kim contributed equally to this work. They are co-first authors.

The authors apologize for this error and state that this does not change the scientific conclusions of the article in any way. The original article has been updated.

