# Supplementary material for: Virally Mediated Connexin 26 Expression in Postnatal Scala Media Significantly and Transiently Preserves Hearing in Connexin 30 Null Mice
Source: Front Cell Dev Biol. 2022 Apr 27;10:900416. doi: 10.3389/fcell.2022.900416 (PMC9091169; doi:10.3389/fcell.2022.900416)
Supplement: Supplementary file 1 [file DataSheet1.docx]

Supplementary Material

# Supplementary Figures and Tables

##
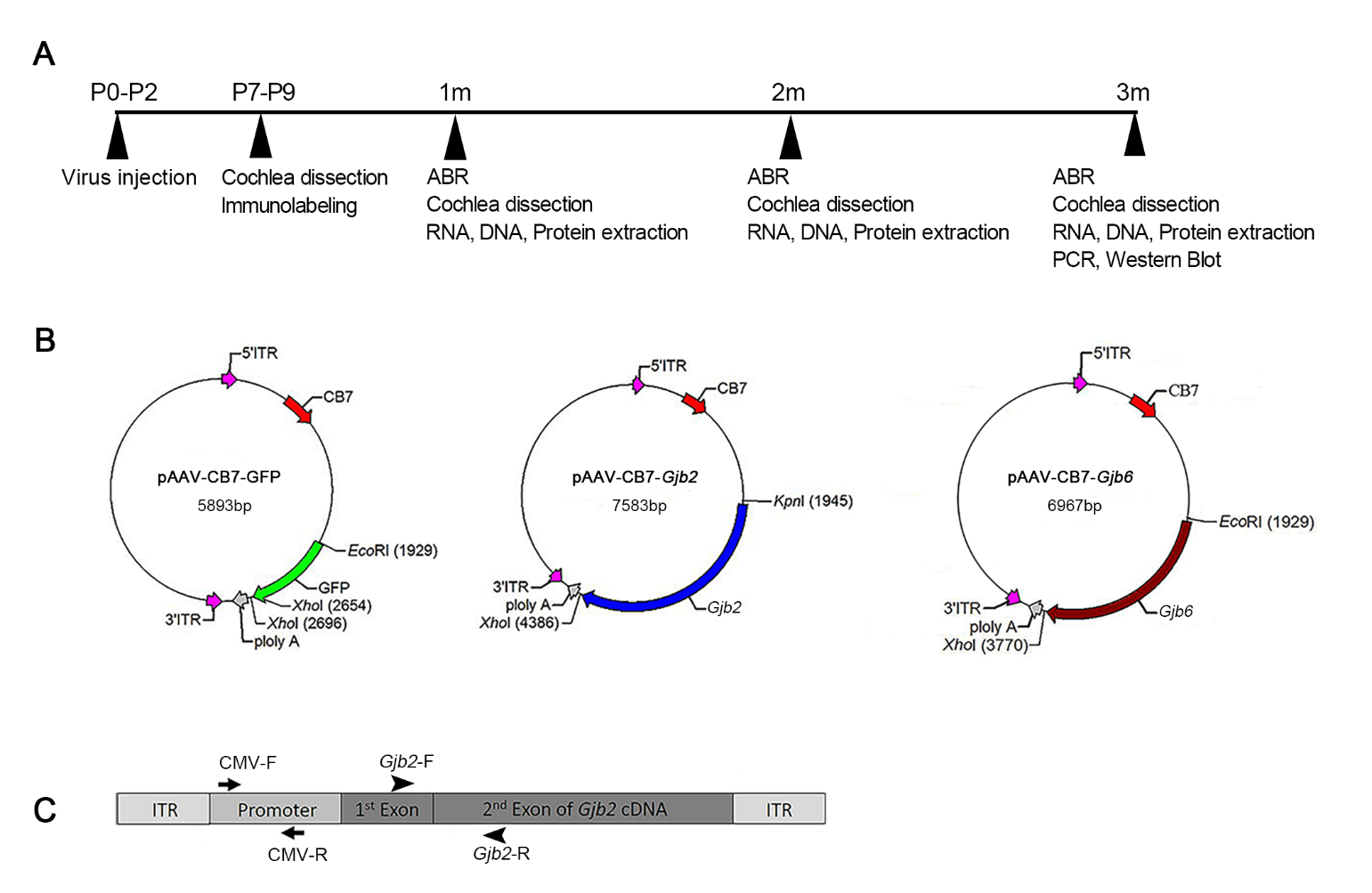
Supplementary Figures

**Supplementary Figure 1. Diagrams illustrating experimental protocols, designs of viral plasmids and the location of primers used to measure viral titer. (A)** Diagram showing the protocol for experiments in gene delivery and functional evaluations of *Gjb6^−/−^* mice. **(B)** Schematic map of plasmids (as labeled) AAV-CB7-GFP, AAV-CB7-*Gjb2* and AAV-CB7-*Gjb6*. **(C)** Schematic outline showing the location of primers used to quantify for viral titer (CMV-F and CMV-R, arrows) and for *Gjb2* mRNA (*Gjb2*-F and *Gjb2*-R, arrow heads) used for semi-qPCR.

**
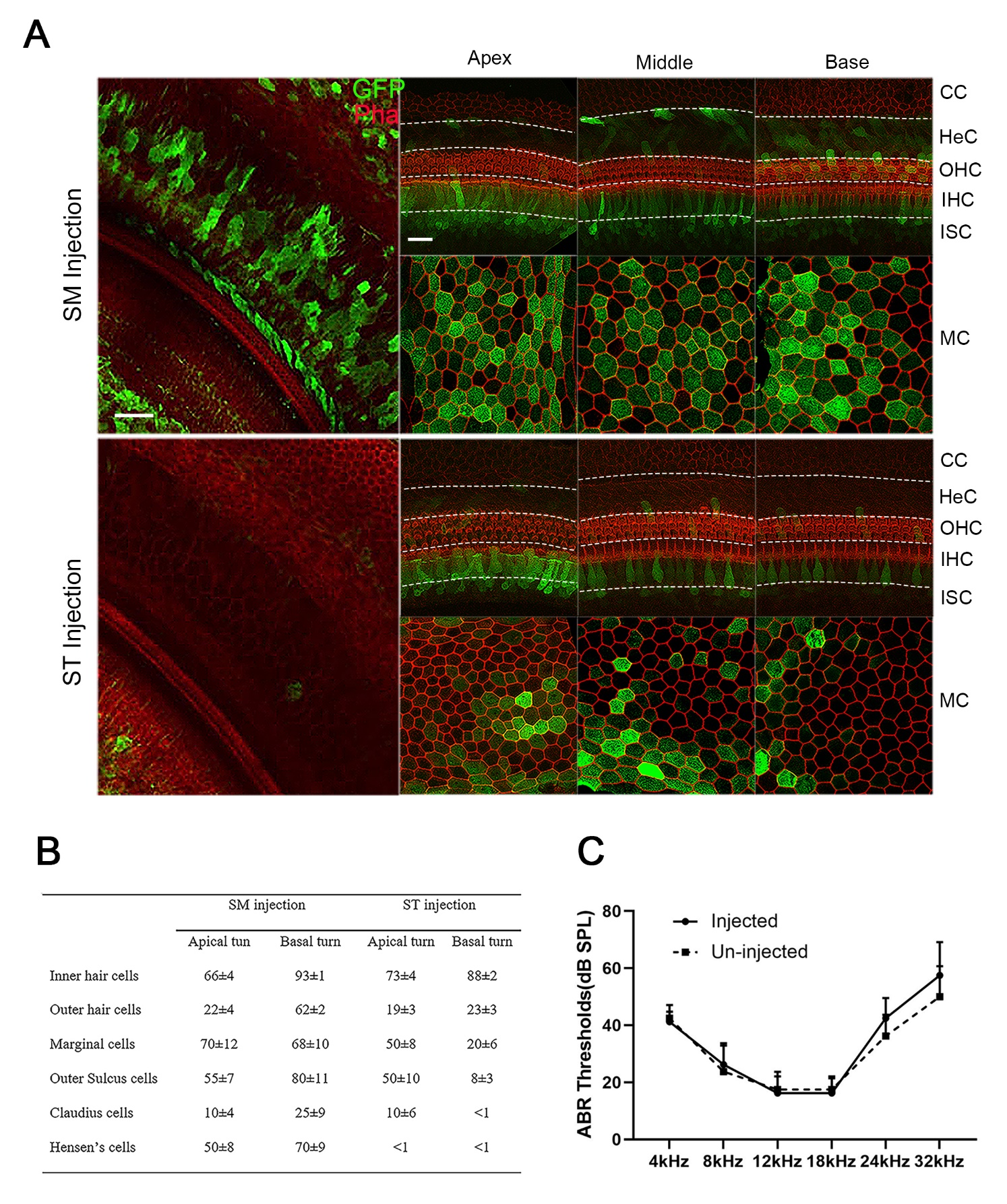
**

**Supplementary Figure 2. Comparison of transduction efficiency between two routes of injections into the SM and ST using AAV1-CB7-GFP.** **(A)** Representative confocal images showing transduction of AAV1-CB7-GFP vector by injections into either SM or ST (as labelled at left of panels). The AAV1-CB7-GFP vector was inoculated into the cochlea of WT mice at P2. The cochlear samples were dissected and stained for GFP at P9. **(B)** Quantification of transduction efficiency as measured by the number of GFP-positive cells. Data are presented as mean ± SD. N=3 for each group. **(C)** Comparison of averaged ABR thresholds of WT mice either injected (at P2, filled circles, n=8) or not injected (filled squares, n=8) with the AAV1-CB7-GFP. Error bars in **C** represent standard deviation. CCs, Claudius cell; HeC, Hensen’s cell; ISC, inner sulcus cell; IHC, inner hair cell; OHC, outer hair cell; MC, marginal cell; Pha, phalloidin. Scale bar: 50µm (low magnification), 20µm (high magnification).

**
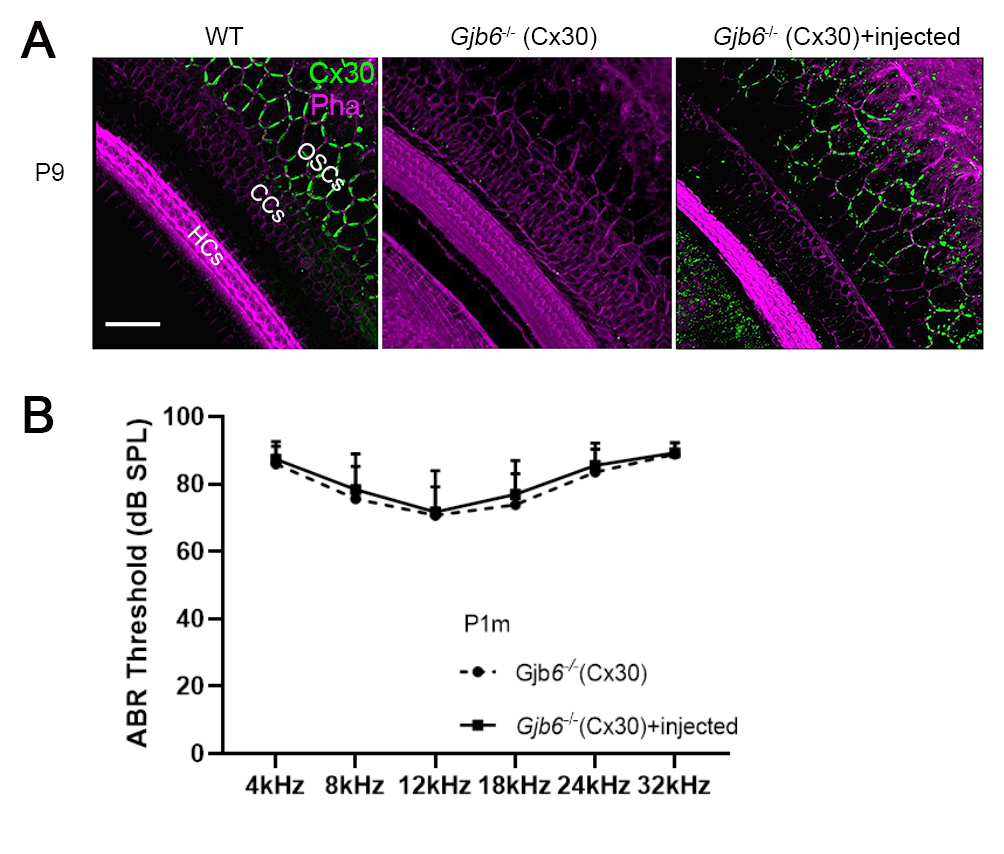
**

**Supplementary Figure 3. Injections of AAV1-CB7-*Gjb6* into the SM expressed Cx30 and apparently increased the number of GJs, but the hearing of *Gjb6^−/−^* mice was not preserved.** (**A**) Representative confocal images of Cx30 expression in the cochlea of WT (left panel), *Gjb6^−/−^* mice without injection (middle panel) and *Gjb6^−/−^* mice injected with AAV1-CB7-*Gjb6* (right panel). (**B**) Comparison of averaged tone burst ABR thresholds measured at P0-2 for un-injected *Gjb6^−/−^* mice (filled circles, n=108) and *Gjb6^−/−^* mice injected with the AAV1-CB7-*Gjb6* (filled square, n=108). Error bars represent standard deviation. OSCs, outer sulcus cells; CCs, Claudius cells; HCs, hair cells; Pha, phalloidin; Cx30, connexin 30. Scale bar: 50µm.

## Supplementary Table

**Supplementary Table 1.** Summary of major features of cochlear gene therapy studies with hearing or vestibular function preservation.

| **Models** | **Vectors** | **Injection time** | **Routes** | **Duration efficacy** |
| --- | --- | --- | --- | --- |
| *Vglut3*^-/-^ mice (Akil et al., 2012) | AAV1-VGLUT3 | P1-3; P10-12 | RWM & Cochleostomy | 7w to 1.5 years |
| *Kcnq1*^-/-^ mice (Chang et al., 2015) | AAV1-CB7-*Kcnq1* | P0-P2 | SM | 18w |
| *MsrB3*^-/-^ mice (Kim et al., 2016) | rAAV2/1-*MsrB3-GFP* | E12.5 | transuterine approach into otocyst | 7w |
| *Whrn* ^wi/wi^ mice (Isgrig et al., 2017) | AAV8-whirlin | P1∼5 | PSCC | 4m |
| *Clrn1* KO-TgAC1 mice (Geng et al., 2017) | AAV2-*Clrn1*-UTR AAV8-*Clrn1*-UTR AAV2-*Clrn1* AAV8-*Clrn1* | P1-3 | RWM | 90 days without UTR; 150 days with UTR |
| *Ush1c c.216G>A* knock-in mice (Pan et al., 2017) | AAV2/Anc80L65. CMV.harmonin-b1 | P0–1 | RWM | 3m |
| *Bth/+* mice (Gao et al., 2018) | Cas9:Tmc1-mut3 sgRNA:lipid | P1 | SM | 8w |
| *Tmc1^Bth/+^* mice (Yoshimura et al., 2019) | AAV9.miTmc1 | P15-16; P56-60 | RWM+CF | 8w after injections |
| *Tmc1*^∆/∆^ mice (Nist-Lund et al., 2019) | AAV2/Anc80L65-*Cmv-Tmc1ex1-WPRE* | P1-2 | RWM | 6-12w |
| *Slc26a4^∆/∆^* and *Slc26a4 ^tm1Dontuh/tm1Dontuh^* mice (Kim et al., 2019) | rAAV2/1-*Slc26a4*-*tGFP* | E12.5 | transuterine approach into otocyst | 3-11w |
| *Slc26a4^−/−^* mice (Takeda et al., 2019) | Plasmid *Slc26a4-Egfp* | E11.5 | EUGO | 90 days |
| *Baringo* mice (Yeh et al., 2020) | Dual AAV-AID-BE3.9max + sgRNA1 | P1 | Inner ear injection | 4w |
| *Syne4*^-/-^ mice (Taiber et al., 2021) | AAV9-PHP.B- *Syne4* | P0-1.5 | PSCC | 12w |
| *Myo6^WT/C442Y^* (Xue et al., 2022) | AAV- PHP.eB-SaCas9-KKH-Myo6-g2 | P0-2 | SM | 5m |
| *Otof*^-/-^ mice (Akil et al., 2019) | AAV-Otof NT + AAV-Otof CT | P10; P17; P30 | RWM | At least 20w |
| *Tmc1 ^Bth/WT^* mice (Gyorgy et al., 2019b) | AAV-SaCas9-KKH-gRNA-4.2 | P1 | SM | At least 24w |
| *Strc*^∆/∆^  mice (Shubina-Oleinik et al., 2021) | Dual AAV9-PHP.B-Strc | P1 | Utricle injection | At least 12w |
| *Tcm1*^∆/∆^ mice *Baringo* mice  *Beethoven* mice (Wu et al., 2021) | AAV9-PHP.B-TCM1 AAV9-PHP.B-TCM1 AAV9-PHP.B-SpCas9 and -gRNA | P1 and P7 P1 and P7 P1 | Utricle injection | At least 12w At least 4w At least 24w |
| Cx30 deficient mice (Miwa et al., 2013) | Plasmid-Cx30 | E11.5 | EUGO | Unclear |
| Cx26^fl/fl^P0-Cre (Iizuka et al., 2015) | AAV1-CMV-Cx26 | P0 | RWM | unclear |
| *Tmc1*^∆/∆^ and *Tmc1*-*Bth* mice (Askew et al., 2015) | AAV2/1-*Cba-Tmc1;* AAV2/1-*Cba-Tmc2* | P0–2 | RWM | unclear |
| *Lhfpl5*^-/-^ mice (Gyorgy et al., 2017) | exo-AAV1-HA-*Lhfpl5* | P1-2 | RWM | unclear |
| *Otof*^-/-^ mice (Al-Moyed et al., 2019) | Dual-AAV-TS; dual-AAV-Hyb | P6-7 | RWM | unclear |
| *Clrn1*^-/-^ mice (Gyorgy et al., 2019a) | AAV9-PHP.B-*Clrn1* | P1 | RWM | unclear |

RWM, round window membrane; SM, scala media; PSCC, posterior semicircular canal; RWM+CF, RWM injection combined with semi-circular canal fenestration; EUGO, Electroporation-mediated transuterine gene transfer into otocysts; w, week; m, month

**References**

Akil, O., Dyka, F., Calvet, C., Emptoz, A., Lahlou, G., Nouaille, S., et al. (2019). Dual AAV-mediated gene therapy restores hearing in a DFNB9 mouse model. *Proc Natl Acad Sci U S A* 116(10)**,** 4496-4501. doi: 10.1073/pnas.1817537116.

Akil, O., Seal, R.P., Burke, K., Wang, C.S., Alemi, A., During, M., et al. (2012). Restoration of Hearing in the VGLUT3 Knockout Mouse Using Virally Mediated Gene Therapy. *Neuron* 75(2)**,** 283-293. doi: 10.1016/j.neuron.2012.05.019.

Al-Moyed, H., Cepeda, A.P., Jung, S., Moser, T., Kugler, S., and Reisinger, E. (2019). A dual-AAV approach restores fast exocytosis and partially rescues auditory function in deaf otoferlin knock-out mice. *EMBO Mol Med* 11(1). doi: 10.15252/emmm.201809396.

Askew, C., Rochat, C., Pan, B., Asai, Y., Ahmed, H., Child, E., et al. (2015). Tmc gene therapy restores auditory function in deaf mice. *Sci Transl Med* 7(295)**,** 295ra108. doi: 10.1126/scitranslmed.aab1996.

Chang, Q., Wang, J., Li, Q., Kim, Y., Zhou, B., Wang, Y., et al. (2015). Virally mediated Kcnq1 gene replacement therapy in the immature scala media restores hearing in a mouse model of human Jervell and Lange-Nielsen deafness syndrome. *EMBO Mol Med* 7(8)**,** 1077-1086. doi: 10.15252/emmm.201404929.

Gao, X., Tao, Y., Lamas, V., Huang, M., Yeh, W.H., Pan, B., et al. (2018). Treatment of autosomal dominant hearing loss by in vivo delivery of genome editing agents. *Nature* 553(7687)**,** 217-221. doi: 10.1038/nature25164.

Geng, R., Omar, A., Gopal, S.R., Chen, D.H., Stepanyan, R., Basch, M.L., et al. (2017). Modeling and Preventing Progressive Hearing Loss in Usher Syndrome III. *Sci Rep* 7(1)**,** 13480. doi: 10.1038/s41598-017-13620-9.

Gyorgy, B., Meijer, E.J., Ivanchenko, M.V., Tenneson, K., Emond, F., Hanlon, K.S., et al. (2019a). Gene Transfer with AAV9-PHP.B Rescues Hearing in a Mouse Model of Usher Syndrome 3A and Transduces Hair Cells in a Non-human Primate. *Mol Ther Methods Clin Dev* 13**,** 1-13. doi: 10.1016/j.omtm.2018.11.003.

Gyorgy, B., Nist-Lund, C., Pan, B., Asai, Y., Karavitaki, K.D., Kleinstiver, B.P., et al. (2019b). Allele-specific gene editing prevents deafness in a model of dominant progressive hearing loss. *Nat Med* 25(7)**,** 1123-1130. doi: 10.1038/s41591-019-0500-9.

Gyorgy, B., Sage, C., Indzhykulian, A.A., Scheffer, D.I., Brisson, A.R., Tan, S., et al. (2017). Rescue of Hearing by Gene Delivery to Inner-Ear Hair Cells Using Exosome-Associated AAV. *Mol Ther* 25(2)**,** 379-391. doi: 10.1016/j.ymthe.2016.12.010.

Iizuka, T., Kamiya, K., Gotoh, S., Sugitani, Y., Suzuki, M., Noda, T., et al. (2015). Perinatal Gjb2 gene transfer rescues hearing in a mouse model of hereditary deafness. *Hum Mol Genet* 24(13)**,** 3651-3661. doi: 10.1093/hmg/ddv109.

Isgrig, K., Shteamer, J.W., Belyantseva, I.A., Drummond, M.C., Fitzgerald, T.S., Vijayakumar, S., et al. (2017). Gene Therapy Restores Balance and Auditory Functions in a Mouse Model of Usher Syndrome. *Mol Ther* 25(3)**,** 780-791. doi: 10.1016/j.ymthe.2017.01.007.

Kim, M.A., Cho, H.J., Bae, S.H., Lee, B., Oh, S.K., Kwon, T.J., et al. (2016). Methionine Sulfoxide Reductase B3-Targeted In Utero Gene Therapy Rescues Hearing Function in a Mouse Model of Congenital Sensorineural Hearing Loss. *Antioxid Redox Signal* 24(11)**,** 590-602. doi: 10.1089/ars.2015.6442.

Kim, M.A., Kim, S.H., Ryu, N., Ma, J.H., Kim, Y.R., Jung, J., et al. (2019). Gene therapy for hereditary hearing loss by SLC26A4 mutations in mice reveals distinct functional roles of pendrin in normal hearing. *Theranostics* 9(24)**,** 7184-7199. doi: 10.7150/thno.38032.

Miwa, T., Minoda, R., Ise, M., Yamada, T., and Yumoto, E. (2013). Mouse otocyst transuterine gene transfer restores hearing in mice with connexin 30 deletion-associated hearing loss. *Mol Ther* 21(6)**,** 1142-1150. doi: 10.1038/mt.2013.62.

Nist-Lund, C.A., Pan, B., Patterson, A., Asai, Y., Chen, T., Zhou, W., et al. (2019). Improved TMC1 gene therapy restores hearing and balance in mice with genetic inner ear disorders. *Nat Commun* 10(1)**,** 236. doi: 10.1038/s41467-018-08264-w.

Pan, B., Askew, C., Galvin, A., Heman-Ackah, S., Asai, Y., Indzhykulian, A.A., et al. (2017). Gene therapy restores auditory and vestibular function in a mouse model of Usher syndrome type 1c. *Nat Biotechnol* 35(3)**,** 264-272. doi: 10.1038/nbt.3801.

Shubina-Oleinik, O., Nist-Lund, C., French, C., Rockowitz, S., Shearer, A.E., and Holt, J.R. (2021). Dual-vector gene therapy restores cochlear amplification and auditory sensitivity in a mouse model of DFNB16 hearing loss. *Sci Adv* 7(51)**,** eabi7629. doi: 10.1126/sciadv.abi7629.

Taiber, S., Cohen, R., Yizhar-Barnea, O., Sprinzak, D., Holt, J.R., and Avraham, K.B. (2021). Neonatal AAV gene therapy rescues hearing in a mouse model of SYNE4 deafness. *EMBO Mol Med* 13(2)**,** e13259. doi: 10.15252/emmm.202013259.

Takeda, H., Miwa, T., Kim, M.Y., Choi, B.Y., Orita, Y., and Minoda, R. (2019). Prenatal electroporation-mediated gene transfer restores Slc26a4 knock-out mouse hearing and vestibular function. *Sci Rep* 9(1)**,** 17979. doi: 10.1038/s41598-019-54262-3.

Wu, J., Solanes, P., Nist-Lund, C., Spataro, S., Shubina-Oleinik, O., Marcovich, I., et al. (2021). Single and Dual Vector Gene Therapy with AAV9-PHP.B Rescues Hearing in Tmc1 Mutant Mice. *Mol Ther* 29(3)**,** 973-988. doi: 10.1016/j.ymthe.2020.11.016.

Xue, Y., Hu, X., Wang, D., Li, D., Li, Y., Wang, F., et al. (2022). Gene editing in a Myo6 semi-dominant mouse model rescues auditory function. *Mol Ther* 30(1)**,** 105-118. doi: 10.1016/j.ymthe.2021.06.015.

Yeh, W.H., Shubina-Oleinik, O., Levy, J.M., Pan, B., Newby, G.A., Wornow, M., et al. (2020). In vivo base editing restores sensory transduction and transiently improves auditory function in a mouse model of recessive deafness. *Sci Transl Med* 12(546). doi: 10.1126/scitranslmed.aay9101.

Yoshimura, H., Shibata, S.B., Ranum, P.T., Moteki, H., and Smith, R.J.H. (2019). Targeted Allele Suppression Prevents Progressive Hearing Loss in the Mature Murine Model of Human TMC1 Deafness. *Mol Ther* 27(3)**,** 681-690. doi: 10.1016/j.ymthe.2018.12.014.
